# Transient Reversal of Episome Silencing Precedes VP16-Dependent Transcription during Reactivation of Latent HSV-1 in Neurons

**DOI:** 10.1371/journal.ppat.1002540

**Published:** 2012-02-23

**Authors:** Ju Youn Kim, Angelo Mandarino, Moses V. Chao, Ian Mohr, Angus C. Wilson

**Affiliations:** 1 Department of Microbiology, New York University School of Medicine, New York, New York, United States of America; 2 Via Manzoni, Viagrande, Italy; 3 Molecular Neurobiology Program, Skirball Institute for Biomolecular Medicine and Departments of Cell Biology, Physiology and Neuroscience, Psychiatry and Center for Neural Science, New York University School of Medicine, New York, New York, United States of America; Wistar Institute, United States of America

## Abstract

Herpes simplex virus type-1 (HSV-1) establishes latency in peripheral neurons, creating a permanent source of recurrent infections. The latent genome is assembled into chromatin and lytic cycle genes are silenced. Processes that orchestrate reentry into productive replication (reactivation) remain poorly understood. We have used latently infected cultures of primary superior cervical ganglion (SCG) sympathetic neurons to profile viral gene expression following a defined reactivation stimulus. Lytic genes are transcribed in two distinct phases, differing in their reliance on protein synthesis, viral DNA replication and the essential initiator protein VP16. The first phase does not require viral proteins and has the appearance of a transient, widespread de-repression of the previously silent lytic genes. This allows synthesis of viral regulatory proteins including VP16, which accumulate in the cytoplasm of the host neuron. During the second phase, VP16 and its cellular cofactor HCF-1, which is also predominantly cytoplasmic, concentrate in the nucleus where they assemble an activator complex on viral promoters. The transactivation function supplied by VP16 promotes increased viral lytic gene transcription leading to the onset of genome amplification and the production of infectious viral particles. Thus regulated localization of *de novo* synthesized VP16 is likely to be a critical determinant of HSV-1 reactivation in sympathetic neurons.

## Author Summary

Herpes simplex virus is a widespread human pathogen that establishes permanent infections in nerves innervating the lips, eyes and other surfaces. The viral DNA genome is transported to the neuronal nucleus located in the nerve ganglia, where it establishes a semi-dormant state known as latency. Periodically, latent viruses undergo reactivation, a process that leads to the production of infectious particles, allowing for person-to-person transmission and acting as the major source for painful lesions (cold sores) and other more severe pathological outcomes. How latency and reactivation are controlled is not well understood. Using cultured nerve cells, we show that reactivation involves a unique, two stage program of viral gene expression. We find that the essential control protein VP16 is synthesized during the first stage but accumulates in the cytoplasm rather than the nucleus where it functions. Nuclear entry is determined by host signaling and marks the onset of the second reactivation stage. This work provides important new insights into the virus-host interaction and reveals a natural control point that could be used in innovative therapies that for the first time target the latent virus.

## Introduction

The remarkable success of the herpesviruses as infectious agents stems from their ability to alternate between productive (acute) replication and latency; distinct genetic programs that achieve very different outcomes for both the virus and the host cell. Acute replication results in release of infectious particles by cell lysis and produces a strong immunological stimulus, whereas in latency the lifespan of the host cell is often extended and the viruses use various strategies to minimize antigen presentation. By alternating between these two programs, herpesviruses can often remain in their host indefinitely but at the same time retain the ability to spread through reactivation, a process whereby latent virus reenters the productive replication cycle and infectious particle are shed at the surface.

The prototype example for this successful strategy is herpes simplex virus type-1 (HSV-1), a widespread human pathogen that infects epithelial cells in the oral cavity, eyes and other regions of mucosa. Latency is established in the ganglia of peripheral nerves that innervate these sites, creating a lifelong reservoir that is shielded from immune clearance (reviewed in [1], [2]). Intermittent reactivation events give rise to infectious particles that travel to the periphery by anterograde axonal transport. Continuous reemergence of virus from the permanent neuronal reservoir ensures lifelong transmission and is often associated with clinical disease.

How the HSV-1 regulates the transition from one program to the other is not well understood. Latent genomes reside in the nucleus of the host neuron as extra-chromosomal circles that are assembled into chromatin resembling that of the host [3], [4]. Transcription is limited to the latency-associated transcripts (LATs) that are spliced into a stable 1.5 to 2.0-kb intron and processed into several microRNAs [5], [6], [7]. The rest of the viral transcriptome corresponding to 80 or so genes is effectively silenced. Although the details are incomplete, it appears that lytic gene transcription is blocked by a combination of mechanisms involving histone modifications that create a repressive chromatin structure, recruitment of factors that prevent the assembly of pre-initiation complexes and the absence of activators need to stimulate RNA polymerase II recruitment and elongation [3], [4]. Chromatin immunoprecipitation (ChIP) studies have shown that the histones associated with the promoter regions of the key regulatory genes carry modifications such as trimethylated histone H3 lysine-9 (H3K9me3) and lysine-27 (H3K27me3) that are typical of facultative heterochromatin [8], [9]. Also present are polycomb (PcG) repressor subunits that may promote chromatin compaction and limit RNA polymerase elongation. Additional layers of repression are also imposed by the REST/coREST silencing complex that recruits the histone deacetylases HDAC1 or 2 and the demethylase LSD1 [10], [11], [12]. When latent episomes undergo reactivation, this heterochromatic signature is replaced with chromatin that is permissive for transcription, enabling the expression of the full repertoire of lytic genes needed to build new infectious virus and neutralize host defenses [13].

Despite intense study by many laboratories there is still much to be learned about the molecular processes that maintain the latent program and orchestrate the transition back into productive replication. Studies have been hampered by the very small numbers of infected neurons in ganglia obtained from human cadavers or animal models, our limited understanding of the signaling pathways that connect natural reactivation stimuli to the latent episome and difficulties in manipulating HSV-infected ganglia in order to test the roles of cellular factors implicated in the process. To circumvent these issues we have taken advantage of a robust cell culture model of HSV-1 latency that uses primary sympathetic neurons isolated from the superior cervical ganglia (SCG) of unborn rats [14]. Homogenous neuron cultures are maintained in the presence of nerve growth factor (NGF) and HSV-1 latency can be established with wild type virus by infection in the presence of acyclovir (ACV). Lytic gene products and infectious virus disappear within a few days but the viral genomic DNA (average of c.25 copies/neuron) remains and LAT RNA can be readily detected in the nuclei of >20% of the neurons by fluorescent *in situ* hybridization, signifying the presence of latent virus. At this stage, ACV can be removed and cultures maintained for up to five weeks without reactivation [15]. Latency is dependent on continuous NGF signaling and interruption of the TrkA/PI3-kinase/Akt branch of the pathway leads to reactivation [14].

This study focuses on the program of viral lytic gene transcription that initiates after NGF signaling is interrupted. Classic studies in non-neuronal cell types have shown that acute (lytic) HSV-1 replication follows an ordered gene expression cascade that can be divided into three temporal phases: immediate-early (IE, α), early (E, β) and late (L, γ) [16], [17]. During low multiplicity infections, the IE phase is initiated by the tegument protein VP16 (also termed α-TIF, UL48, vmw65), which collaborates with cellular factors to drive IE gene transcription [18], [19], [20]. The IE proteins then act as positive regulators of the E genes, leading to amplification of the HSV-1 DNA genome and transcription of the L genes, which encode structural components of the viral particle, including the tegument protein VP16. Whether reactivation follows the same transcription program is uncertain, not least because the context of the viral genome is radically different in the two situations. At the onset of reactivation, the viral DNA is incorporated into ordered chromatin consisting of regularly spaced nucleosomes, whereas at the beginning of acute infection cycle the DNA is essentially nucleosome-free. Additionally, regulatory proteins such as VP16 that incorporated into the virion tegument are almost certainly absent at the beginning of reactivation.

Using pharmacological inhibition of PI3-kinase to achieve synchronous reactivation, we find that viral lytic mRNAs accumulate in two discrete waves (dubbed Phase I and II) that differ in the need for viral protein synthesis, DNA replication and the initiator factor VP16. In Phase I, lytic transcripts of all three kinetic classes accumulate simultaneously. New viral protein synthesis is not required, consistent with a short-term reversal of episome silencing. Genome amplification begins in Phase II and is followed by infectious virus production. VP16 accumulates in the cytoplasm during Phase I but most interestingly, does not contribute to viral transcription until Phase II, when nuclear VP16 is first detected. During acute infections, VP16 assembles an activator complex on viral promoters in the nucleus through interactions with host factors Oct-1 and HCF-1 [21], [22], [23], and providing human Oct-1, which has a higher affinity for VP16 than its rodent counterparts, to the neurons increases viral transcript levels during Phase II, arguing for the assembly of an equivalent VP16-induced complex. We conclude that reactivation follows a unique program of gene expression initiated by a transient reversal of host-mediated gene silencing and suggest that regulated entry of VP16 into the neuronal nucleus represents a host-imposed barrier that must be overcome for successful viral reactivation to occur.

## Results

### Sequential cascade of viral mRNA accumulation during acute replication in sympathetic neurons

**Figure 1:**
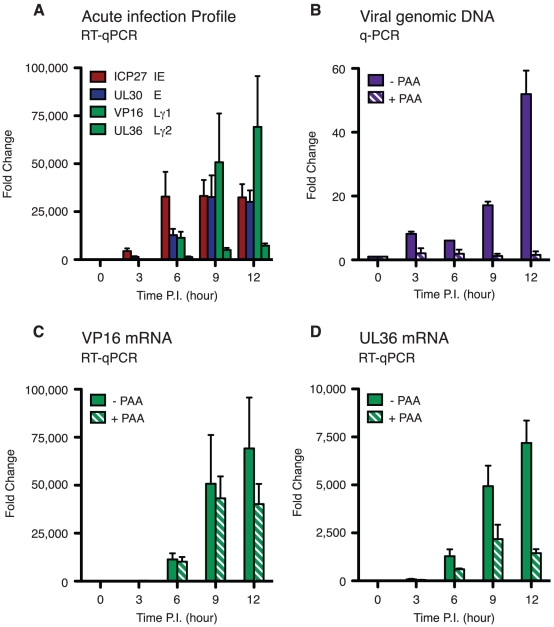
Acute replication of HSV-1 in SCG neurons follows the canonical ordered cascade of mRNA accumulation. (A) Primary neurons were isolated from superior cervical ganglia (SCG) of E21 rats, cultured for 7 days in the presence of 5 µM aphidicolin and 20 µM 5-fluorouracil to eliminate proliferating cells, and then infected with HSV-1 GFP-Us11 at a multiplicity of 3 plaque forming units per neuron (MOI = 3). RNA was collected at 0, 3, 6, 9, and 12 h post-infection (p.i.) and analyzed by quantitative reverse transcription PCR (qRT-PCR) to determine the relative levels of viral immediate-early (ICP27), early (UL30), γ1 leaky-late (VP16) and γ2 true-late (UL36) transcripts. Values represent the average and standard error from the mean from three independent infection experiments. (B) Neuron cultures were treated with the viral DNA polymerase inhibitor phosphonoacetic acid (PAA, 300 µg/ml) for 1 h (hatched bars) or mock treated (filled bars) and then infected with HSV-1 GFP-Us11. Total DNA was prepared at the indicated times and the relative levels of viral genomic DNA determined by quantitative (qPCR) using primers complementary to the HSV-1 UL30 gene. Input DNA was normalized by qPCR detection of the rat RPL19 gene. (C, D) Analysis of γ1 leaky-late (VP16) and γ2 true-late (UL36) transcript levels in the presence or absence of PAA.

As a prelude to our analysis of reactivation in sympathetic neurons, we profiled viral mRNA accumulation during acute replication (Figure 1). Week-old neuron cultures were infected with recombinant HSV-1 GFP-Us11 at a multiplicity of infection of 3 PFU/neuron (MOI = 3). Under these conditions, the virus replicated productively and the majority of neurons expressed the GFP-Us11 fusion protein within the first day. RNA was collected at intervals between 0 and 12 h of infection and analyzed by quantitative reverse transcription PCR (qRT-PCR) using primers specific to lytic transcripts representing each kinetic class: IE (ICP27/UL54), E (DNA polymerase/UL30), leaky-late/γ1 (VP16/UL48) and true-late/γ2 (tegument phosphoprotein ICP1-2/UL36). The first transcripts were detected within 3 h and became increasingly abundant at later time points. Importantly, we found that the accumulation of these mRNAs followed the classic IE-E-L profile (Figure 1A).

To pinpoint the onset of viral genome amplification, total DNA was also collected at each time point and assayed by qPCR using primers complimentary to a region in the UL30 gene (Figure 1B). Aside from a small increase in DNA content between 0 and 3 h that likely reflects the binding and uptake of viral particles into the neurons, robust amplification of the viral genome began between 6 and 9 h post-infection. This correlates with the appearance of transcripts from UL36 (Figure 1A and 1D), which is defined as a replication-dependent (true-late or γ2) gene [24]. To confirm that the increase in genome content reflected virus-directed replication, the time course was repeated in the presence of phosphonoacetic acid (PAA), an inhibitor of the virus-encoded DNA polymerase (Figure 1B). As expected, there was no increase in DNA content in the presence of the inhibitor, even at 12 h. By definition, leaky-late (γ1) and true-late (γ2) genes are distinguished by the degree of sensitivity to PAA and other inhibitors of viral DNA synthesis. Accordingly, VP16 mRNA (γ1) showed only a limited decrease in the presence of PAA (Figure 1C), whereas the levels of UL36 mRNA (γ2) were significantly decreased (Figure 1D). Expression of GFP-Us11 also conforms to true-late kinetics and was sensitive to PAA (data not shown). Thus, productive HSV-1 infection in SCG-derived neurons follows a sequential program of gene expression that mirrors the classic lytic cascade defined in non-neuronal cells. In agreement with an earlier study using SCG neurons [25], we find that the duration of the lytic program is 2 to 3 times longer in primary neurons than in non-neuronal cells such as rat embryo fibroblasts (Figure S1).

### Viral mRNAs accumulate in two discrete phases during reactivation

Ex vivo studies of reactivation have relied on the analysis of latently infected ganglia that are surgically axotomized from the animal and then explanted into culture. Under these circumstances the neurons are severely stressed and it is likely that multiple reactivation triggers occur. Alternatively, reactivation can be induced by physiological stress prior to analysis (discussed in [26]). After explant, viral lytic mRNAs are synthesized very rapidly, making it difficult to establish the order of events and limiting the opportunities for manipulating the neuronal environment by non-pharmacological means. Mindful of these issues, we turned to the SCG neuron culture model as a tractable experimental system to study the first stages of reactivation. Treatment of latently-infected SCG cultures with the phosphatidyl inositol-3 kinase (PI3-K) inhibitor LY294002, is sufficient to activate latent HSV-1 episomes, resulting in the expression of a lytic reporter (GFP-Us11) in 15–20% of the neurons within 48 h [14]. This provides a single, defined reactivation stimulus that simplifies subsequent analyses.

**Figure 2:**
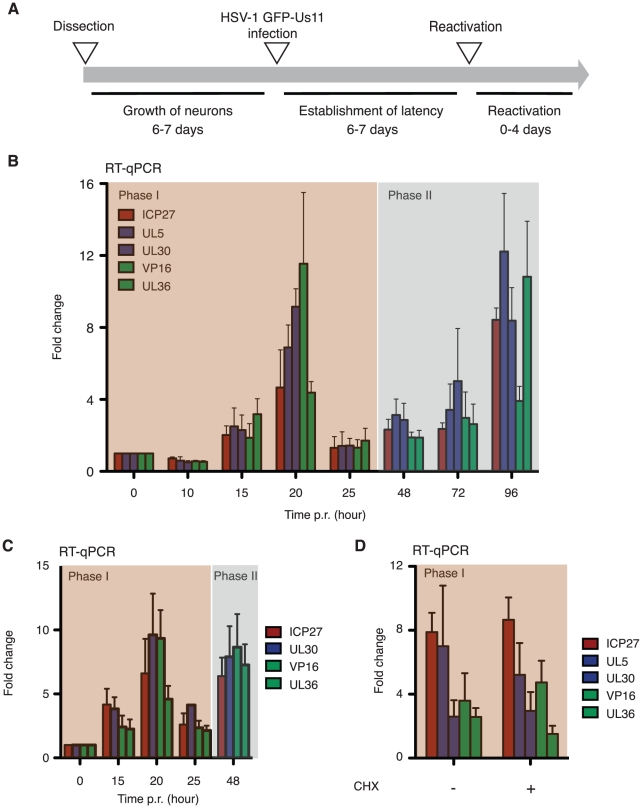
During reactivation, HSV-1 exhibits a biphasic profile of viral transcripts in SCG neurons. (A) Scheme showing a typical reactivation experiment. Neuron cultures were established and then infected with HSV-1 GFP-Us11 (MOI = 1) in the presence of 100 µM acyclovir (ACV). Latency was established over a 7-day period before re-feeding with fresh media lacking ACV. The next day, reactivation was induced with 20 µM LY294002. (B) Profile of viral mRNA accumulation in response to LY294002. RNA was collected at the indicated times and analyzed by qRT-PCR. Values are normalized against the 0 h sample [ICP27, 171 copies/sample; UL5, 135 copies/sample; UL30, 94 copies/sample; VP16, 347 copies/sample and UL36 130 copies/sample]. Data is derived from three or more independent cultures and reactivation experiments. (C) Reactivation profiling in the presence of the viral DNA encapsidation inhibitor WAY150138 (20 µg/ml). (D) Transcript levels at 20 h post induction in the absence (−) or presence (+) of protein synthesis inhibitor cyclohexamide (CHX, 10 µg/ml). To ensure cell viability, CHX was added 10 h after LY294002, prior to the appearance of new viral transcripts.

Latently infected cultures were established over a one-week period in the presence of 100 µM acyclovir (ACV) to suppress lytic replication and then reactivated following the scheme outlined in Figure 2A. Once latency was established, fresh media containing LY294002 and lacking ACV was added to induce reactivation. After inducing reactivation, total RNA was collected at successive time points over a four-day period and analyzed by qRT-PCR using viral primer sets normalized to 18S rRNA (Figure 2B). A baseline for each lytic mRNA was established from samples collected when the inducer was first applied (abundance ranges from 94 copies/sample for UL30 to 347 copies/sample for VP16) and all other values are expressed relative to these low but measurable levels. The analysis revealed a protracted time course of lytic mRNA accumulation compared to the acute infection studies (see Figure 1A), with a strong but transient accumulation of IE, E and L viral mRNAs at 20 h, followed by a decline between 20 and 25 h and then a gradual accumulation from 48 to 96 h, the last time point. The analysis shown summarizes the data from three independent experiments prepared from separate neuron preparations made on different dates. Mock treatment with DMSO resulted in no reproducible change in baseline levels over a 72 h period (data not shown). In all of these experiments, and in others not shown here, we observed a similar protracted profile corresponding to a discernable spike in lytic mRNA accumulation at 15 to 20 h post-induction, a striking decrease at around 25 h and then a second, more gradual, rise at later times. This profile is clearly very different from the rapid and sequential accumulation of lytic mRNAs observed during acute infection of similar cultures. We will refer to these two discrete periods or waves of lytic mRNA accumulation as Phase I and Phase II.

A simple explanation for the biphasic profile is that a limited number of (primary) reactivation events produce infectious virus that subsequently spreads to neighboring cells and replicate further as (secondary) acute infections. We ruled out this out by including WAY-150138, a stable compound that prevents encapsidation of the DNA genome and blocks spread of infectious virus [27], [28]. Repeating the reactivation time course in presence of WAY-150138 produced a very similar, biphasic profile (Figure 2C), indicating that secondary infection was not responsible for the second peak of lytic mRNA accumulation.

It is known that brief exposure to the protein synthesis inhibitor cycloheximide (CHX) is sufficient to induce HSV-1 reactivation in both SCG and dorsal root ganglion neuron cultures [29]. To ask whether the synthesis of viral proteins is required for Phase I, we induced reactivation with LY249002 first and then maintained the cultures for 10 h to allow the earliest reactivation steps to proceed before CHX (10 µg/ml) was added. At this time, viral lytic transcripts remained at base line levels making it highly unlikely that any viral proteins had been synthesized. RNA was collected after a further 10 h in the presence of CHX (i.e. at 20 h post-induction with LY249002) and analyzed by qRT-PCR (Figure 2D). When compared to mock treated cultures, no significant changes were observed in the levels of lytic mRNAs, indicating that Phase I initiates normally in the absence of viral protein synthesis.

**Figure 3 ppat-1002540-g003:**
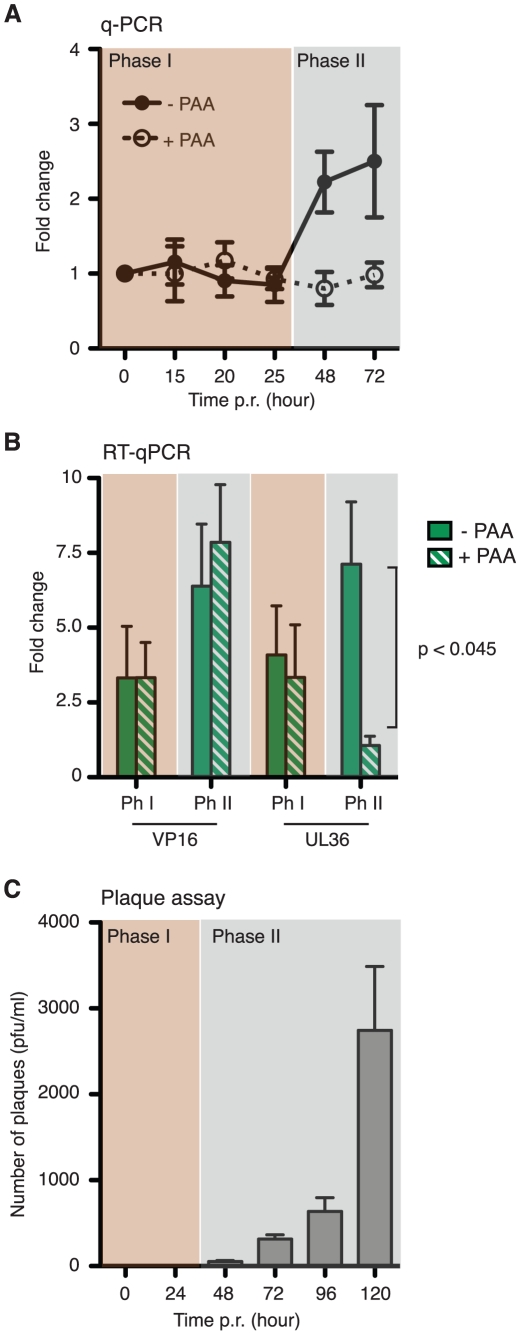
HSV-1 GFP-Us11 replication occurs in Phase II of reactivation. Latent cultures of SCG cells were induced with LY294002 as described before. (A) Viral DNA Content was determined by qPCR at different times post-reactivation (p.r.) in the absence or presence of 300 µg/ml PAA. (B) Quantitative RT-PCR analysis of VP16 and UL36 mRNA levels in the absence or presence of 300 µg/ml PAA during Phase I (15–20 h) and in Phase II (48 h). (C) Number of infectious virus particles was determined by plaque assay.

Next we sought to determine when viral genome amplification begins. DNA was collected at successive intervals and analyzed by qPCR (Figure 3A). Under these assay conditions we can reliably detect an increase in viral DNA content of 15% or greater (data not shown). No increase in viral DNA content was observed until the 48-h time point, which corresponds to Phase II and was not seen when the experiment was repeated in the presence of the PAA. The lack of demonstrable amplification during Phase I (15 and 20 h post induction) at least within the limits of the assay, was especially interesting given that we could readily detect representative late gene transcripts at 20 h. To examine this more closely, we compared the expression of UL36 mRNAs in the presence or absence of PAA at 20 h (Figure 3B). No significant difference was observed, showing that accumulation of UL36 mRNA in Phase I was replication-independent. By contrast, inclusion of PAA decreased the accumulation of UL36 mRNA during Phase II in a reproducible and significant manner (P-value<0.045).

We also performed plaque assays to detect infectious virus particles (Figure 3C). Acknowledging the sensitivity issues associated with this biological assay [27], we were unable to detect plaques until 48 h post LY294002 treatment, with a significant increase in plaque number from 72 h onwards. This suggests that infectious virus is not assembled until well into Phase II, concomitant with viral genome amplification and reinforces the notion that the transition from Phase I to Phase II does not reflect secondary infection.

### Reactivation requires the transactivation function of VP16

**Figure 4:**
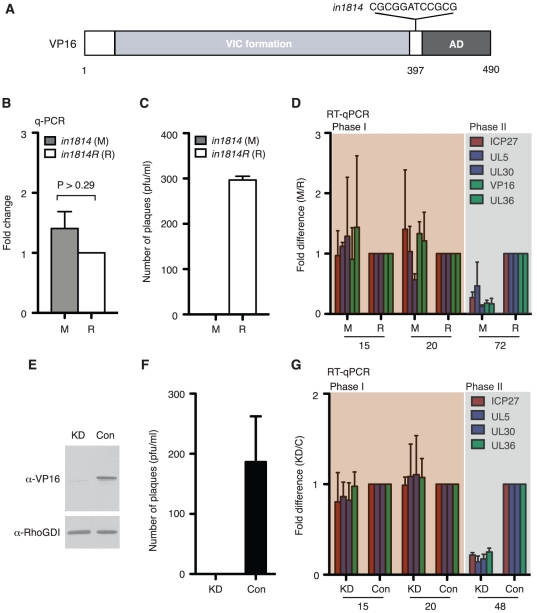
Transactivation function of VP16 is required during Phase II. (A) Structure of VP16 showing the 12-bp insertion (*in*1814) between the structured N-terminal domain and the C-terminal activation domain (AD) that disrupts VP16-induced complex assembly [32]. (B) SCG neurons were infected with mutant (*in*1814) or marker rescue (*in*1814R) viruses (MOI = 1) in the presence of 100 µM acyclovir and maintained for 7 days before measuring the relative amounts of viral genomic DNA by qPCR. (C) Reactivation was induced with 20 µM LY294002 in media lacking ACV and maintained for 7 days before harvest and plaque assay to detect infectious virus. (D) Comparison of viral transcript levels during reactivation by *in*1814 (‘M’) and *in*1814R (‘R’) at 15 and 20 h post-induction (Phase I) and at 72 h post-induction (Phase II). For each time point, transcript levels from the *in*1814 (‘M’) sample were set to 1 and the value for the corresponding transcript from *in*1814R (‘R’) plotted as the fold difference. (E) Depletion of VP16 using RNA interference. Latently infected neuron cultures were infected with a lentivirus expressing a VP16-specific short-hairpin RNA [shRNA] (KD) or with a control lentivirus (Con). ShRNAs were allowed to accumulate for 5 days before reactivation was induced with LY294002 and allowed to proceed for 5 days in media lacking ACV. Lysates were prepared and probed by immunoblotting to detect VP16 and the loading control, Rho-GDI. (F) Quantitation of infectious virus by plaque assay. (G) Comparison of viral transcript levels in the absence of VP16. Values from the control culture are plotted relative to the corresponding value from the VP16 shRNA (KD) culture.

VP16 is essential for HSV-1 replication, functioning in the assembly of the tegument layer and as a potent transactivator of the IE genes, which in turn drive the expression of the E genes [30], [31]. Recombinant viruses have been characterized that carry mutations in VP16 that preserve the essential tegument forming function but selectively impair the transactivation function, either by preventing the formation of the VP16-induced complex on DNA response elements found in each IE promoter [32] or by removing the C-terminal transactivation domain [33], [34]. Previous work has shown these mutants can still establish latency in animal models, and depending on the reactivation assay used, can display severe defects in reactivation [35], [36]. To examine the contribution of VP16 to LY294002-induced reactivation in the SCG system, we performed infection studies using *in*1814, a derivative of strain 17*syn* ^+^ that contains a 12-bp insertion at codon 397 (Figure 4A) [32]. Neuron cultures were infected in the presence of ACV with equal titers (MOI = 1) of *in*1814 or a repaired version (*in*1814R) that behaves similar to wild type strain 17*syn* ^+^ and allowed to establish latency. To obtain equivalent titers each virus stock was grown on complementing human U2OS cells and then plaque assayed on rat embryo fibroblasts in the presence of 5 mM hexamethylene bisacetamide (HMBA), a differentiation agent that overcomes the requirement for transactivation by VP16 during productive replication at low multiplicity [37]. The *in*1814 and *in*1814R viruses established latency at a similar frequency as judged by quantitation of viral genomic DNA after six days in the presence of ACV (Figure 4B). Slightly more genomes were detected with *in*1814, however, this difference was not statistically significant (P>0.29). The ability of *in*1814 to establish latency mirrors its behavior in several latency models [33], [35], [36], [38], [39]. Next, latently infected cultures were induced with LY294002 and the yield of infectious virus was determined by plaque assay on Vero cells in the presence of HMBA (Figure 4C). In spite of the similar numbers of genome at the time of induction, we observed a profound difference in the yield of infectious virus. Indeed, over multiple experiments we were unable to detect a single infectious particle for *in*1814. This indicates that loss of the transactivation function of VP16 has a highly deleterious effect on the ability of HSV-1 to reactivate successfully in cultured neurons following a specific stimulus. This agrees with previous studies using the murine hyperthermal stress model for reactivation [36].

### VP16 contributes to reactivation Phase II but not Phase I

To better understand the nature of the *in*1814 reactivation defect, we measured the relative levels of viral mRNAs produced by the mutant and rescued viruses in each reactivation phase (Figure 4D). For ease of comparison, values for *in*1814 are shown relative to the corresponding *in*1814R samples and the entire profile for mRNA accumulation from *in*1814 is shown in Figure S2A. When the two viruses are compared at 15 or 20 h post-induction (Phase I), no significant differences were observed, however at 72 h (Phase II) all five representative lytic mRNAs were expressed at approximately 5-fold lower levels by *in*1814 compared to *in*1814R (Figure 4D). Likewise, no amplification of the *in*1814 genome was detected (Figure S2B), consistent with the inability of the mutant virus to produce infectious progeny. Thus we conclude that the transactivation function of VP16 contributes to lytic gene transcription during the Phase II only, and that reduced expression of viral proteins results in a failure to replicate the genome. This would account for the absence of infectious progeny following reactivation.

### Reduction of Phase II transcript levels using VP16 shRNA

An important caveat in using mutant viruses to study reactivation is the possibility that the mutations alter some aspect of the establishment of latency that subsequently impacts reactivation. To circumvent this concern and independently validate the striking phenotype of the *in*1814 virus, we developed a short-hairpin RNA (shRNA) capable of depleting newly synthesized VP16 from wild type virus. We have previously shown that latently infected SCG neurons can be successfully infected with lentiviruses without inducing reactivation and that shRNAs delivered in this manner are effective at depleting endogenous proteins [14]. The ability to deplete viral products in this system was previously untested. Neurons were latently infected with the HSV-1 GFP-Us11, allowed to establish latency and then secondarily infected with lentiviruses encoding either the VP16 shRNA or a non-silencing control. The vector includes a constitutive mCherry marker and visual inspection confirmed that the majority of neurons were infected (data not shown). After 5 days of dual infection, LY294002 was added to the media and reactivation allowed to proceed. VP16 depletion was confirmed by qRT-PCR to detect VP16 mRNA (Figure S2C) and by immunoblot of lysates prepared at 5 days post-induction (Figure 4E). VP16 is required for tegument assembly and accordingly, infectious virus was not detected in cultures expressing the VP16 shRNA, corroborating the knockdown (Figure 4F). More importantly, we compared the levels of lytic transcripts at 15 and 20 h (Phase I) and 48 h (Phase II). As before, we observed no significant difference in Phase I, but saw a 4 to 5-fold reduction in each of the lytic mRNAs in Phase II (Figure 4G). This result confirms the phenotype of the *in*1814 virus and underscores the importance of VP16-mediated transactivation during Phase II.

### Human Oct-1 increases levels of viral mRNA in Phase II

**Figure 5:**
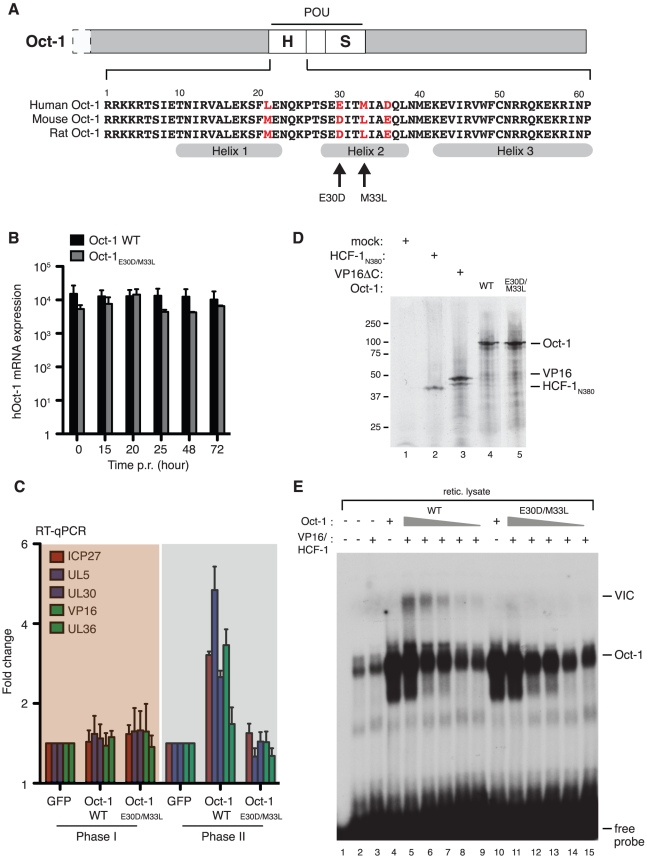
Elevated Phase II viral transcript levels in neurons expressing human Oct-1. (A) Schematic of Oct-1 showing the location of the POU DNA-binding domain near the middle of the protein and an alignment of the human, mouse and rat POU homeo (POU_H_) subdomain sequences. The four variable positions are located in helix-1 and helix-2 and are numbered according to their position within the POU_H_ sequence. A rodent-like Oct-1 derivative (Oct-1_E30D/M33L_) was constructed by changing glutamic acid-30 and methionine-33 to the aspartic acid and leucine of the mouse/rat sequence. (B) SCG neuron cultures were infected with HSV-1 GFP-Us11, maintained for 5 days in the presence of ACV and then infected with lentiviral vectors encoding GFP or human Oct-1. After a further 5 days, ACV was removed and reactivation induced with 20 µM LY294002. RNA was collected at intervals and analyzed by qRT-PCR using human Oct-1 specific primers. For each time point, values for each transcript were compared to those from the GFP-expressing neurons (set to 1.0). (C) Relative levels of viral transcripts (ICP27, UL5, UL30, VP16 and UL36) after reactivation of HSV-1 GFP-Us11 in neurons expressing GFP, wild type human Oct-1 (WT) and human Oct-1_E30D/M33L_ (MUT). (D) Wild type (WT) and E30D/M33L (MUT) versions of human Oct-1, VP16 (residues 5–412, VP16ΔC) and the β-propeller domain of human HCF-1 (residues 1–380, HCF-1_N380_) were synthesized by in vitro translation in the presence of ^35^S-methionine and visualized by 10% SDS-PAGE followed by autoradiography. (E) Assembly of the VP16-induced complex (VIC) by rodent-like (E30D/M33L) Oct-1 is greatly reduced compared to wild type human Oct-1. Recombinant Oct-1, VP16 and HCF-1 proteins were assayed for VIC formation by gel shift assay using a ^32^P-labeled probe containing an (OCTA^+^)TAATGARAT element from the HSV-1 ICP0 promoter [23]. The first three lanes are controls showing probe alone (lane 1), un-programmed rabbit reticulocyte lysate (lane 2), and a mix of lysates containing recombinant VP16 and HCF-1 (lane 3). The shift formed by the rabbit Oct-1 present in the lysate is greatly enhanced by the presence of either wild type or mutant human Oct-1 (lanes 4 and 10). A slower migrating complex (VIC) is formed by addition of VP16 and HCF-1 in the presence of wild type Oct-1 (lane 5) and only weakly by the mutant (lane 11). Reducing the amount of wild type Oct-1 by 5, 10, 50 and 100-fold respectively (lanes 6–9) reduces but does not eliminate this complex. No VIC is detected over a similar range of using mutant Oct-1.

VP16 is recruited to the TAATGARAT sequences found in each HSV-1 IE promoter through physical association with HCF-1 and the POU domain transcription factor Oct-1 [30]. Selection of Oct-1 (also called POU2F1) is highly specific and the presence of other POU proteins may actually antagonize the VP16-induced complex by competing for the TAATGARAT sites. The Oct-1 POU DNA-binding domain is sufficient for VP16-induced complex formation, with VP16 recognizing a solvent exposed surface on the POU-homeo (POU_H_) subdomain [40]. Although the amino acid sequence of the POU domain has been conserved through vertebrate evolution (Figure 5A), there are four differences between the POU_H_ sequences of human and either rat or mouse Oct-1 [41]. As a consequence, VP16 has a significantly lower affinity for rodent Oct-1 and reduced ability to drive transcription from reiterated TAATGARAT elements in murine 3T3 cells [42]. Whether this difference between rat and human Oct-1 has an equivalent effect on the activity of the IE promoters in the context of the reactivating virus is unknown. To address this, we used a lentiviral expression vector to introduce human Oct-1 into rat SCG neurons that were already latently infected with HSV-1 Us11-GFP. After the 5-day establishment period, separate cultures were transduced with either a lentivirus expressing wild type human Oct-1 or a control lentivirus expressing GFP. After a further 5 days, reactivation was induced with LY294002 and RNA samples prepared at regular intervals. Effective delivery of the Oct-1 lentivirus was confirmed by qRT-PCR analysis using human Oct-1 specific primers (Figure 5B). Amplification products were readily detected in the samples containing the Oct-1 expression lentivirus but not with the control virus and the levels remained essentially unchanged through the time course of the experiment. Analysis of viral lytic mRNAs performed in parallel revealed the expected biphasic profile in both sets (data not shown). During Phase I (15 to 20 h) no significant differences in transcript levels were observed between neurons expressing human Oct-1 or GFP, but in Phase II viral mRNA levels were increased of up to 5-fold in the presence of human Oct-1 (Figure 5C).

To determine whether this increase was simply a consequence of providing more Oct-1, we performed parallel infections with a human Oct-1 derivative in which the two non-conserved residues on helix-2 (corresponding to POU_H_ sequence glutamic acid-30 and methonine-33, highlighted in Figure 5A) were swapped to the rodent equivalent (aspartic acid-30 and leucine-33). These two residues are reported to have the greatest impact on VP16 binding [42] and the deleterious effect of the combined mutation was confirmed by gel-shift assay using in vitro translated Oct-1, VP16 and HCF-1 proteins (Figure 5D, E). Both wild type (lane 4) and mutant human Oct-1 (lane 10) bind with similar efficiency to a ^32^P-labeled probe containing an (OCTA^+^)TAATGARAT sequence from the HSV-1 ICP0 promoter [23]. As expected, wild type Oct-1 supports VP16-induced complex (VIC) formation (lanes 5–9) but this is reduced by 100-fold or greater with the E30D/M33L mutation (lanes 11–15). Efficient lentiviral infection for the mutant was evident by a strong qRT-PCR signal using human Oct-1 primers (Figure 5B) although levels were slightly lower than with wild type. Importantly, we observed no significant change in viral lytic mRNA levels in the Oct-1 E30D/M33L transduced cultures, either in Phase I or in Phase II compared to the control lentivirus expressing GFP (Figure 5C), indicating that the stimulation observed by wild type human Oct-1 in Phase II is most likely due to improved interactions with VP16. We conclude from this experiment that the transactivation function provided by VP16 in Phase II requires Oct-1, presumably through the formation of a multi-protein complex on the TAATGARAT elements found in each IE promoter.

### VP16 is expressed in Phase I but remains in the cytoplasm

**Figure 6:**
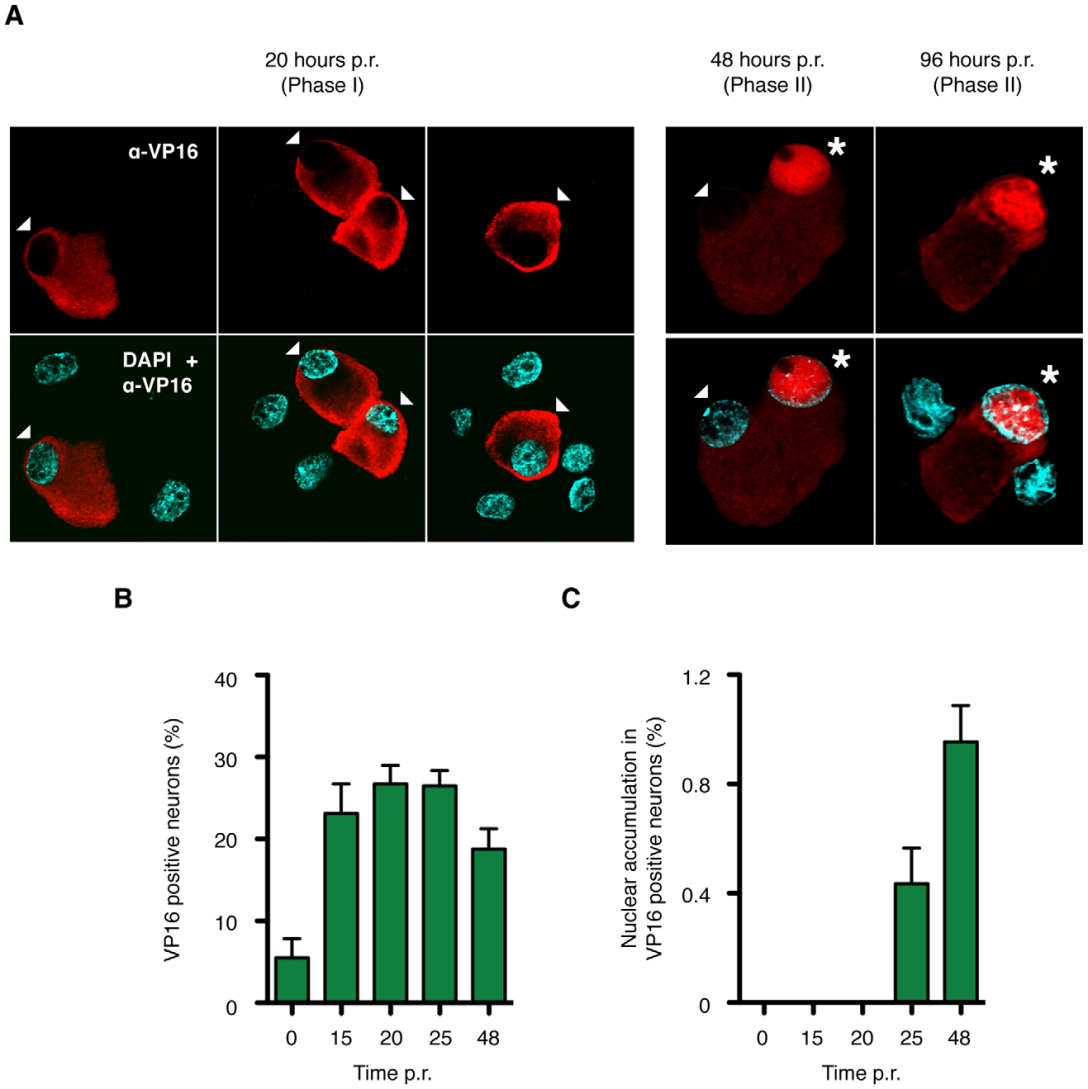
VP16 is expressed during Phase I but is localized to the cytoplasm. (A) Detection of VP16 in latently infected neurons by indirect immunofluorescence microscopy using an α-VP16 rabbit polyclonal antibody (red). Individual nuclei were visualized with DAPI (blue). Three representative fields are shown from cover slips collected at 20 h post-induction (Phase I) and single fields from 48 and 96 h (Phase II). An arrowhead is used to indicate the cell body of neurons with a predominantly cytoplasmic VP16 signal, whereas those displaying a strong nuclear VP16 signal are indicated with an asterisk. Neurons that do not express VP16 are left unmarked but are evident from the DAPI stained nuclei. (B) Quantitation of the immunofluorescence analysis. Numbers of VP16 positive neurons are expressed as a percentage of all neurons scored. Data is compiled from three independent reactivation experiments. Between 500 and 3,000 neurons were scored per cover slip. (C) Percentage of VP16 positive neurons with a predominantly nuclear rather than cytoplasmic α-VP16 signal.

Although the transactivation function of VP16 is dispensable for Phase I, VP16 mRNA is readily detected (Figures 2–4). This prompted us to ask when the VP16 protein is synthesized. To do this we performed indirect immunofluorescence microscopy on latently infected neurons undergoing reactivation (Figure 6). Using a rabbit polyclonal antibody against VP16, we detected a strong signal in approximately 5% of the neurons at the onset of reactivation (0 h, see discussion), which rose to more than 20% of the culture at 15 and 20 h, which corresponds to Phase I (Figure 6A and 6B). The signal corresponds to VP16 because the antibody recognizes a single species of the correct size on immunoblots (Figure 4E), is absent from the remaining 80% or more neurons in the cultures (Figure 6A) and can be suppressed or eliminated by coinfection with the VP16 shRNA lentivirus (data not shown).

Strikingly, at 0 h and later time points the VP16 signal was preferentially localized to the soma (cell body) of the neurons rather than the nucleus (indicated by arrow heads and counterstained with DAPI), an unexpected distribution for a transcription factor. By contrast IE proteins ICP0 and ICP27 were detectable as speckles in the nucleus during Phase I showing that multiple viral antigens are expressed at this time and that nuclear uptake was not inhibited (Figure S3). The cytoplasmic VP16 signal persisted through the Phase I–II transition (25 h) and into Phase II (48 and 96 h), however most interestingly, a small but increasing number of neurons showed a predominantly nuclear VP16 signal at 25 and 48 h (Figures 6A and 6C). The nuclear signal appeared diffuse throughout the nucleoplasm and was excluded from the single large nucleolus characteristic of neurons. This distribution is consistent with a general association with chromatin, rather than formation of discrete viral replication compartments within the nucleus [43].

### Localization of VP16 cofactor HCF-1 is regulated by PI3-kinase inhibition

**Figure 7:**
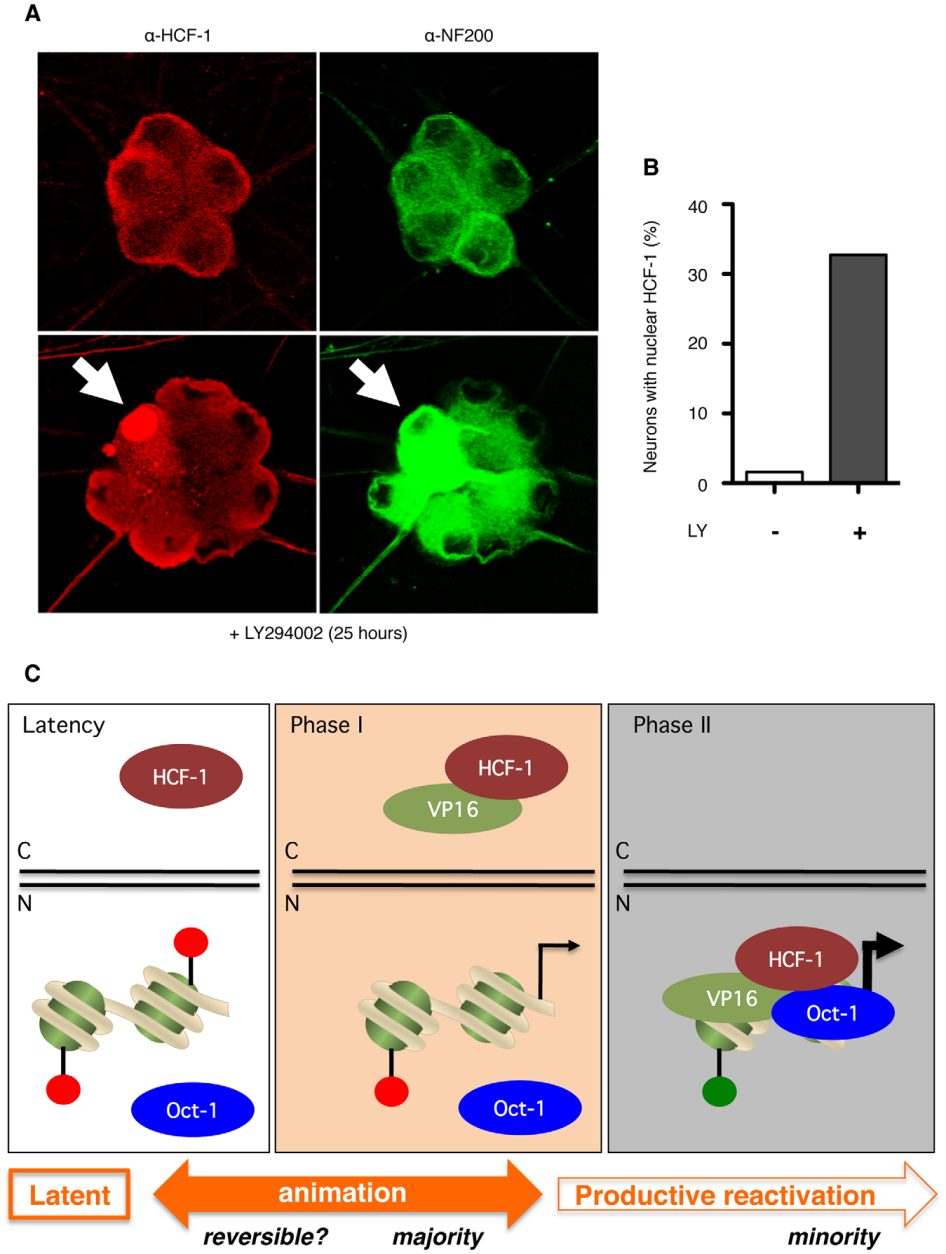
Relocalization of HCF-1 in response to PI3-kinase inhibition. (A) Confocal image showing a cluster of neuronal cell bodies probed with α-HCF-1 rabbit polyclonal antibody (red) and α-neurofilament-200 (NF200) mouse monoclonal antibody (green) at 25 h post treatment with LY294002 (lower panels) or vehicle (upper panels). Note HCF-1 signal throughout the cell body and axonal processes with sparing in the nucleus. Nuclear accumulation is detected in one neuron (arrow) in the LY294002-treated sample. (B) Quantitation of neurons showing obvious nuclear HCF-1 at 25 h post treatment with LY294002 (+) or vehicle (−) (C) Model for the two-step reactivation program observed in sympathetic neurons induced by inhibition of NGF signaling. The latent HSV-1 episome resides in the nucleus (‘N’) and is associated with histones carrying post-translational modifications typical of repressed chromatin (red lollipops). HCF-1 is sequestered in the cytoplasm, whereas Oct-1 is presumed to be nuclear throughout. Host-mediated alterations to episome chromatin during Phase I allows for generalized transcription of viral lytic genes. VP16 is synthesized, but accumulates in the cytoplasm (‘C’), possibly through association with HCF-1. We speculate that a smaller number of animation events advance to Phase II, coincident with the accumulation of VP16 and HCF-1 into the nucleus permitting the assembly of the VP16-induced complex on viral IE promoters in association with Oct-1. This leads to the recruitment of additional chromatin modifiers that apply chromatin marks associated with active transcription (green lollipops).

HCF-1 is nuclear in most cell types with the notable exception of sensory neurons, where it accumulates in the cytoplasm [44]. Ganglia explant and other stresses, trigger relocalization of HCF-1 into the nucleus, where it can associate with HSV-1 IE gene promoters [44], [45]. In light of the dynamic localization of VP16 in SCG neuron cultures, we also probed for HCF-1 using a polyclonal antibody directed against the HCF-1_C_ subunit (Figure 7A). The immunofluorescence signal is widely dispersed throughout the cytoplasm including the axons and dendrites (upper panels). Nuclear sparing is clearly evident and the cytoplasmic HCF-1 did not appear to co-localize with major cytoplasmic organelles. When cultures are treated with LY294002 for 25 h, we saw significant accumulation of nuclear HCF-1 in some but not all of the neurons (Figure 7A, lower panels). Counting revealed a strong nuclear signal in more than 30% of the LY294002-treated neurons (n = 293) compared to less than 2% in mock-treated cultures (n = 1244) (Figure 7B). Control studies using antibodies to the HCF-associated proteins Ash2L [46] and LSD-1 [11] revealed predominantly nucleoplasmic staining with obvious nucleolar sparing, irrespective of treatment (Figure S4). Thus we can reproduce the differential and dynamic localization of HCF-1 first described in murine trigeminal ganglia, and show for the first time that HCF-1 localization can be manipulated by the inhibition of a cellular signal transduction pathway critical for the maintenance of HSV-1 latency.

## Discussion

Latent infection of primary SCG neuron cultures provides a powerful system to pick apart the mechanisms that control HSV-1 reactivation in response to defined physiological changes in the host neuron. Viral and cellular regulatory factors can be more readily manipulated in culture than in live-animal models and use of relatively pure populations of neurons allows us to set aside potentially confounding layers of control imposed by HSV-specific CD8^+^ T cells and other cell types resident in the ganglia. Thus analyses can be focused on the intimate and fundamental relationship between the latent virus and it's host neuron. Using this reductionist approach, we have profiled the transition from the latent to lytic transcription programs after treating latently infected cultures with a selective PI3-kinase inhibitor that interrupts NGF signaling [14], [29]. In contrast to acute infections, we find that lytic mRNAs accumulate in two discrete waves - termed Phase I and Phase II - that differ profoundly in their requirement for viral protein synthesis and DNA replication (see model in Figure 7C). Accumulation of viral transcripts during Phase I was unaffected by addition of the protein synthesis inhibitor CHX prior to the onset of viral mRNA accumulation, indicating that changes in pre-existing factors may be sufficient to bring about a generalized, but apparently temporary, de-repression of the lytic transcriptome. Studies published while this work was in revision also find that IE, E and L transcripts accumulate concurrently in the presence of CHX following explant-induced reactivation from the trigeminal ganglia of mice infected by corneal inoculation [47]. Our data does not provide evidence for the selective activation of key viral genes and suggests instead that there is activation of a broad spectrum of lytic genes. We acknowledge, however, that only a small fraction of the lytic transcriptome has been surveyed and that exceptions may exist.

While Phase I appears to be unique to the reactivation program, Phase II more closely resembles the ordered cascade seen during acute infection of both neuronal and non-neuronal cells. Lytic mRNA levels are enhanced by the transactivation function of the VP16-induced complex, true-late gene transcription is dependent on viral DNA replication and this phase culminates in the production of infectious virus. From the evidence obtained so far, we favor the notion that Phase I might serve as a ‘priming’ stage from which virus may or may not progress into a ‘production’ or ‘synthesis’ stage (Phase II). In this context, about a quarter of the neurons engage in Phase I as judged by VP16 and ICP27 fluorescence, but it seems likely that a much smaller number advance into Phase II as signified by either the presence of nuclear VP16 (this study) or the accumulation of true-late protein Us11 (Us11-GFP) in the presence of a compound that prevents secondary infection [14]. Further work is needed to establish the exact relationship between entry of VP16 into the nucleus and onset of Phase II-specific processes such as genome amplification and virion assembly.

Initiator protein VP16 is synthesized in Phase I but evidently does not contribute a necessary function until Phase II. Using two strategies we show that in the absence of VP16-mediated transactivation, the second phase of reactivation program proceeds at a reduced level and ultimately does not yield infectious virus. Thus we can conclude that VP16 is essential for reactivation in the SCG model following interruption of NGF signaling. Whether this is the result of insufficient transcription of the IE genes or loss of some less well understood function of VP16 remains to be addressed. A similar requirement for VP16 is observed in vivo during reactivation from murine trigeminal ganglia in response to thermal stress [36] but interestingly is not required for reactivation from the same ganglia after axotomy/explant, which is generally considered a more severe and multifaceted stimulus [35], [48]. This difference between assay approaches highlights the complex nature of the reactivation literature in which a number of different experimental systems have been used to explore the viral and cellular requirements for reactivation [49]. Although different conclusions have been drawn, the results are not necessarily incompatible.

While speculative, the idea of two-stage process for synthesizing and using VP16 (and possibly other viral regulatory factors) is appealing because it mirrors the delivery of tegument proteins including VP16 during acute infections [18], [20]. This strategy would perhaps allow HSV-1 to evaluate the capacity of the host neuron to support virion production, which is both metabolically demanding and involves extensive remodeling of the viral chromatin, before proceeding to point that might threaten the survival of the neuron or render the viral genome vulnerable to innate defenses. Reactivation events that do not meet the necessary criteria for completion of Phase II, for instance if there was insufficient nuclear accumulation HCF-1 and/or VP16, may even retain the option of re-establishing latency. This ability would buffer the virus from wide scale reactivation under moderate stress conditions and thereby help to maintain the latent reservoir. Although the overall amounts of viral mRNA were similar in samples collected during Phase I and Phase II this is unlikely to be a true representation of the transcriptional activity at each stage. Assuming that a smaller number of genomes progress into Phase II, the actual rates of transcription for any given reactivating episome will be much higher, consistent with the strong activator functions associated with VP16 and HCF-mediated remodeling of viral chromatin. In theory, this intriguing idea could be substantiated or refuted by following the progression of individual neurons through the reactivation program.

The transition from a repressed (silenced) chromatin state to one that allows high levels of transcription requires the concerted action of a variety of histone modifying enzymes and chromatin remodeling factors. An active area of study, there is already compelling evidence that HSV-1 uses a combination of viral and cellular factors to overcome silencing imposed by polycomb (PcG) repressor complexes [8], a REST/coREST/LSD1 repressor complex [1], [11] and possibly other mechanisms. ChIP studies of chromatin isolated from infected ganglia have established that core histones associated with the latent episome are enriched for H3K27me3, a repressive mark that is the signature of control by PcG proteins [8]. This and other repressive epigenetic marks are lost during reactivation and replaced with di/trimethylated histone H3 lysine-4 (H3K4me2/3), a mark of active transcription [50], [51]. Future work will determine whether silencing marks are removed at the onset of Phase I or are retained until Phase II, perhaps providing an epigenetic imprint that facilitates re-establishment of the latency program for genomes that do not progress into Phase II.

Exhaustive studies in mammalian cell lines and more heterologous systems such as yeast have shown that the individual components of the VP16-induced complex can recruit a variety of factors that promote active transcription [46], [50], [51], [52]. The well-studied acidic activation domain of VP16 is capable of recruiting or altering the activity of several general transcription factors, the Mediator complex, histone acetyltransferases and ATP-dependent chromatin remodeling factors as well as RNA polymerase II itself (reviewed in [53]). Whether the same processes and interactions apply during reactivation is not known. It is also worth noting that HCF-1 can bind to a variety of cellular transcription factors either directly or indirectly, and that some of these factors have binding sites in the HSV-1 IE and E gene promoters [54]. Moreover, HCF-1 is a component of several coactivators complexes, including the Set1 histone H3K4 methyltransferase complexes that is an active modifier of HSV-1 chromatin during acute infections [52], [55]. Thus it remains a possibility that under some circumstances, the necessary chromatin-modifying activities are recruited to viral chromatin through the association of HCF-1 and cellular DNA binding proteins rather than VP16. This might explain why the transactivation function of the VP16-induced complex is not required under all reactivation circumstances [35], [48].

As our understanding of reactivation advances it is helpful to draw a clear distinction between the overarching process of reactivation that ends with the release of infectious progeny and a more narrowly defined ability of the viral episome to transcribe lytic genes. Feldman and colleagues used ‘spontaneous molecular reactivation’ to describe events in which viral lytic genes are abundantly expressed but no infectious virus is detected [56]. Whether reactivation is curtailed by the action of adjacent immune cells within the ganglia or is intrinsic to the individual neurons to remains to be established. More recently, Penkert and Kalejta applied the succinct term ‘animation’ to describe the initial departure from the latent program prior to the onset of virion production [2]. This strikes us as a good descriptor for the Phase I stage detailed for the first time in this study because it allows for a flexible program in which the virus can either advance to full reactivation or re-establish latency if certain thresholds are not met.

Beyond the mechanistic details, partitioning of the reactivation program into two discrete phases has important implications for the control of HSV-1 latency by the host immune system. In the absence of an applied reactivation stimulus, we found that up to 5% of the neurons in our cultures were VP16 positive by immunofluorescence assay but otherwise the cultures lacked obvious signs of reactivation such as strong GFP-Us11 expression or detectable infectious particles. Although the levels of viral antigen expression seem high compared to previous in vivo studies, this is likely a reflection of the very high colonization rate (25–50% of neurons) in this in vitro system ([14], unpublished studies). In vivo, similar animation events will appear much less frequently because far fewer neurons are colonized however these rare events could still serve as the source of viral antigens detected by HSV-specific CD8^+^ T cells that infiltrate sensory ganglia in latently infected animals [57]. By manipulating the Phase I–II transition, it may be possible to propel a greater number of animation events into active replication phase rendering the virus vulnerable to both cell-mediated immunity and the action of current antiviral compounds. This would offer a new and worthy strategy to target the latent reservoir, a long-sought-after but elusive goal.

## Materials and Methods

### Ethics statement

This study was carried out in strict accordance with the recommendations laid out by the NIH Guide for the Care and Use of Laboratory Animals. A detailed protocol for the isolation of rat primary neurons was approved by the Institutional Animal Care and Use Committee (IACUC # 101009) of the NYU Langone Medical Center (PHS Assurance of Compliance Number: A3435-01). Rats were euthanized by CO_2_ inhalation prior to ganglia harvest.

### Preparation of SCG neuron cultures

Primary neuron preparation, culture and infection was performed using our established procedures [14]. Briefly, superior cervical ganglia (SCG) were isolated from E21 Sprague Dawley® rat pups, placed in Leibovitz's L-15 medium supplemented with 2 mM L-glutamine, 0.4% D-glucose and incubated with 0.1% trypsin at 37°C for 30 min. Next, ganglia were washed in C-medium (minimum essential medium with Earle's salts, 0.4% D-glucose, 10% FBS, 2 mM L-glutamine) and dissociated by passing through 21G and 23G needles and a 70 µm nylon cell strainer. Dissociated cells were seeded in 24-well plates at a density of 4–5×10^4^ cells per well with C-medium supplemented with NGF (50 ng/ml, Harlan Bioproducts). To improve adherence, wells were pre-coated with collagen (0.6 mg/ml, Millipore) and laminin (2 µg/ml, Sigma). The following day, media was changed to NBM (neural basal media, 0.4% D-glucose, 2 mM L-glutamine, 1x B-27) supplemented with NGF and with aphidicolin (5 µM, Calbiochem) and 5-fluorouracil (20 µM, Sigma) to remove proliferating cells.

### HSV-1 infection of SCG neuron cultures

For acute infections, cultures were maintained for 6 days as described above and then incubated with HSV-1 (Patton) GFP-Us11 (MOI = 3) for 2 h at 37°C. Latent infections were established by adding 100 µM acyclovir (ACV, Calbiochem) to the culture media on day 6, infecting the following day with HSV-1 (MOI = 1). After 2 h at 37°C, the media was replaced with NBM with 50 ng/ml NGF and 100 µM ACV and maintained for 7 days to allow latency to establish. Cultures were induced to reactivate with by replacing the media with fresh NBM containing the PI3-kinase inhibitor LY294002 (Calbiochem, 20 µM) and 50 ng/ml NGF but omitting ACV. When using HSV-1 GFP-Us11, individual wells were inspected prior to induction for GFP fluorescence that might be indicative of low level spontaneous reactivation, and discarded if positive. Typically no more than 10–20% of cultures undergo spontaneous reactivation after mock treatment, most likely due to the stress of handling.

### Transcript analysis by quantitative reverse transcription PCR (qRT-PCR)

Total RNA was prepared from neuron cultures using the RNeasy Mini kit (QIAgen) with some minor modifications. After washing with PBS, neurons were lysed in 350 µl RLT Buffer (QRNeasy Mini Kit, QIAgen) and homogenized using QIAshredder (QIAgen). The total volume was brought up to 900 µl with water and treated with 90 µg/ml Protease K for 10 minutes at 55°C. RNA was precipitated with 450 µl of 100% ethanol and applied to columns provided by the kit. Columns were washed as recommended and eluted with RNase-free water. To eliminate contaminating DNA, RNA was treated with DNase I (New England Biolabs) for 30 min at 37°C and the DNase I heat inactivated at 75°C for 10 min in the presence of 2 mM EDTA. RNA concentrations were determined using a NanoDrop spectrophotometer (ThermoScientific) and 300 ng/sample were used to generate cDNA with Superscript III (Invitrogen) and random hexamer primers (Fermentas). Levels of selected viral lytic mRNAs were quantified by qRT-PCR using the following primer sets:

ICP27 FW: 5′-TTTCTCCAGTGCTACCTGAAGG-3′

ICP27 RV: 5′-TCAACTCGCAGACACGACTCG-3′

UL5 FW: 5′-ACGTCGAGCTGTTGTTCGTCCA-3′

UL5 RV: 5′-GGCGAGCGTGCGTTTGATTT-3′

UL30 FW: 5′-CGCGCTTGGCGGGTATTAACAT-3′

UL30 RV: 5′-TGGGTGTCCGGCAGAATAAAGC-3′

VP16 FW: 5′-TCGGCGTGGAAGAAACGAGAGA-3′

VP16 RV: 5′-CGAACGCACCCAAATCGACA-3′

UL36 FW: 5′-CGCTGCACGAATAGCATGGAATC-3′

UL36 RV: 5′-CCAGCTCCCCGGAACACATTTA-3′

Primers were designed against the HSV-1 17*syn* ^+^ strain reference sequence (GenBank NC_001806) using Primer 3 design software [58]. Input RNA was normalized using 18S rRNA primers (SA Biosciences) and the following equation: dCt (threshold cycle) = Target gene Ct- 18s rRNA Ct. Real-time qPCR analysis was performed using FastStart Universal SYBR Green Master-ROX (Roche) and a MyiQ™ single-color real-time thermal cycler (BioRad). Relative changes in transcript levels were calculated using the ΔΔCt method. Data plots and statistical calculations were made using Prism 5.0 software (GraphPad).

### Analysis of viral DNA replication

Neurons were washed in PBS, lysed with DNA extraction buffer (150 mM NaCl, 10 mM Tris pH 8, 10 mM EDTA, 10% SDS) and transferred to microcentrifuge tubes. Lysates were treated with 100 µg/ml Protease K overnight at 55°C, followed by two rounds of phenol extraction, one round of chloroform extraction and ethanol precipitation. DNA was re-suspended in water and analyzed by qPCR using primers to HSV-1 UL30 and normalized to cell number by amplification of rat RPL19 (RPL19 FW 5′-ATGTATCACAGCCTGTACCTG-3′ and RPL19 RV 5′-TTCTTGGTCTCTTCCTCCTTG-3′). To selectively inhibit lytic DNA replication, 0.3 mg/ml phosphonoacetic acid (PAA, Sigma) was added to the media 1 h before acute infection or with LY294002 for reactivation.

### Growth of HSV-1 stocks and titer determination

Viral stocks were amplified on Vero (GFP-Us11) or U2OS (*in*1814, *in*1814R) cells. Semi-confluent monolayers were infected at MOI = 0.01 and maintained at 34°C for 2–3 days before harvest by freeze-thaw lysis and sonication. Infectious titers were determined by plaque assay using either Vero cells or rat embryo fibroblasts. Uninfected cells were seeded at a density of 5×10^5^/well in 6-well plates and incubated with lysate for 2 h at 37°C before being overlaid with 0.5% agarose in MEM supplemented with 1% calf serum. After incubation for 3 days at 37°C, cells were fixed with 10% TCA for 15 min and stained with 1% crystal violet.

### Immunofluorescence microscopy

Neurons were seeded at density of 4–5×10^4^ onto coverslips pre-coated with poly-D lysine, collagen and laminin and later fixed using 4% paraformaldehyde/20% sucrose in PBS for 15 min, quenched with 100 mM ammonium chloride for 5 min and permeabilized with 0.1% Triton X-100 in PBS. After blocking in 1% BSA in PBS for 15 min, coverslips were incubated with polyclonal antibodies against VP16 (α-VP16 pep153, diluted 1∶1,000, a kind gift of Michael Gregory Peterson, Tularik Inc.) for 2 h at room temperature or against HCF-1 (α-rHCF H12 [59], diluted 1∶1,000) and detected using Alexa555-conjugated α-rabbit IgG antibody (diluted 1∶1,000, 1 h at 37°C). Nuclei were stained with 1 µg/ml 4′, 6-diamidino-2-phenylindole (DAPI) and the coverslips were mounted with DakoCytomation fluorescent mounting medium (Dako Corp.). Samples were visualized using a Zeiss LSM 510 META laser scanning confocal microscope. Images were captured with the Zeiss AIM software and exported into Adobe Photoshop 7.0 for cropping and minor adjustments.

### Protein extraction and immunoblotting

Cells were washed in PBS and then lysed in medium salt extraction buffer (250 mM KCL, 20 mM Tris-HCl pH 7.9, 10% glycerol, 0.25% NP40) on ice for 1 h. The supernatant was collected after centrifugation for 10 min at 11,000 rpm at 4°C and fractionated by 10% SDS-PAGE. Separated proteins were transferred onto nitrocellulose membranes and blocked with 5% milk in TBS-T (200 mM Tris, 1.4 M NaCl, 1% Tween) for 30 min at room temperature prior to incubation overnight at 4°C with α-VP16 (diluted 1∶1,000) or α-Rho-GDI (Santa Cruz, diluted 1∶5,000), followed by HRP-conjugated α-rabbit IgG antibody (Roche, diluted 1∶2,500).

### Lentiviral delivery of VP16 shRNA and human Oct-1

Depletion of HSV-encoded VP16 was achieved using shRNA delivered into latently infected neurons by lentivirus (pLVTHM-VP16shRNA). An oligonucleotide duplex 5′-cgcgtccccGAGTGTAAATTCCTATCAATtcaagaGATTGATAGGAATTTACACTCtttttggatccat-3′ containing the hairpin sequence (upper case) was inserted between the unique Mlu I and Cla I sites of a pLVTHM derivative that has been modified to constitutively express mCherry [14]. A human Oct-1 (GenBank NM_002697) cDNA was expressed from lentiviral vector pEZ-Lv105 vector (GeneCopoeia). Altered sense mutations (E30D/M33L) were introduced by successive rounds of QuikChange II XL Site-Directed Mutagenesis (Stratagene) and verified by DNA sequencing. Lentiviral stocks were generated by transfection of HEK 293LTV cells (Cell Biolabs) using calcium phosphate co-precipitation to introduce a mixture of the lentiviral vector plasmid and two packaging plasmids (pΔ8.9 and pMD2.G) in an equal ratio. Precipitates were left on the cells overnight and then washed. A control virus encoding GFP was prepared in the same manner. Supernatants containing lentivirus were collected on days 3 and 4. To infect neuron cultures, 500 µl of lentiviral-containing supernatant was added to each well, incubated overnight and replaced with fresh media the following day. For latently infected cultures, lentivirus was added 5 days after HSV-1 infection in the presence of ACV and reactivation with LY294002 was performed 5 days after lentivirus infection.

### Gel-shift analysis

Recombinant human Oct-1, VP16ΔC and HCF-1_N380_ were synthesized by in vitro translation using the TNT quick-coupled transcription/translation system (Promega) in the presence of Easytag L-[^35^S]-methionine (Perkin Elmer) as previously described [60], [61]. The E30D/M33L mutation was introduced into wild type human Oct-1 by site-directed mutagenesis. A radiolabeled double-stranded DNA probe was prepared by PCR amplification using ^32^P-labeled primers to amplify a subcloned ICP0 (OCTA+)TAATGARAT sequence [23]. Binding reactions containing labeled probe, 1 µg poly-dI-dC, 10 mM Hepes pH 7.9, 75 mM KCl, 1 mM EDTA, 10 mM DTT and up to 4 µl reticulocyte lysate were incubated at room temperature for 20 min and loaded on a 4% native polyacrylamide gel with 1x Tris-glycine-EDTA buffer. Electrophoresis was carried out at room temperature and run at a constant 170 V. Gels were fixed in methanol/acetic acid, dried and the probe visualized by autoradiography using an X-ray film to block the ^35^S signal.

### Accession numbers

The Genbank (http://www.ncbi.nlm.nih.gov/Genbank/) accession number for the HSV-1 17*syn* ^+^ strain reference sequence is NC_001806. Database accession numbers for proteins analyzed in this study are: rat HCF-1 (NM_001139507.1); human Oct-1 (NP_002688); rat Oct-1 (NP_001094109), and VP16 (Swiss-Prot: P04486.2).

## Supporting Information

Figure S1 **Acute replication of HSV-1 in primary rat embryo fibroblasts (REFs).** One day before infection, REFs were seeded into 6-well plates at a density of 2.0×10^5^ cells/well. The next day, HSV-1 GFP-Us11 was added at a multiplicity of 3 (MOI = 3) and incubated at 4°C for 1.5 h to promote synchronous infection. The temperature was then shifted to 37°C (time post infection = 0 h) and RNA and DNA samples were collected at hourly intervals. (A) Quantitative reverse-transcription PCR to determine the relative levels of representative viral lytic transcripts. Because viral transcripts accumulated to very high levels (up to 10^6^-fold increase), the fold change is plotted as a percentage of the maximum value for each primer set over the 7 h period. Values are the mean and standard error of the mean from two independent infection experiments. (B) Quantitative PCR analysis of viral genomic DNA using primers to the UL30 locus. Samples were prepared from synchronous infection in presence or absence of the viral DNA polymerase inhibitor PAA (300 µg/ml). Input DNA was normalized using primers to the neuronal RPL19 gene.
(EPS)

Figure S2 **VP16 contributes to viral gene expression during Phase II.** (A) Time course of viral mRNA accumulation in SCG cultures latently infected with HSV-1 *in*1814 (MOI = 1) and induced to reactivate with 20 µM LY294002 in media lacking ACV. (B) Quantitative PCR analysis of viral genomic DNA of *in*1814 or *in*1814R at 0 or 72 hours in the presence or absence of PAA. (C, D) Five days after infection with HSV-1 GFP-Us11 in the presence of 100 µM acyclovir, SCG neurons were secondarily infected with a lentivirus expressing an shRNA against VP16 (KD) or control lentivirus (Con). After a further 5 days, the neurons were treated with 20 µM LY294002 to induce reactivation. At various times post treatment (15, 20, 48 or 72 h), total RNA was prepared and assayed by RT-qPCR to determine relative amounts of VP16 transcript (C). As levels of VP16 mRNA increased with time, the relative extent of knockdown also increased as shown here by plotting the fold change between the control and VP16 shRNA samples. (D) Viral mRNA accumulation over time course. Data is derived from three independent experiments and values represent the average and standard error of the mean.
(EPS)

Figure S3 **Lytic proteins ICP0 and ICP27 are expressed in Phase I and are localized to the nucleus.** Week old SCG neuronal cultures were infected with either *in*1814 (A) or HSV GFP-Us11 (B) at MOI = 1 in the presence of ACV. After one week of establishing latency period, the cultures were induced with media lacking ACV but containing 20 µM LY294002. Samples were fixed either 25 hours (A) or 20 hours (B) after induction. ICP0 was detected using a monoclonal antibody (Virusys, 1∶100) and ICP27 was detected with a polyclonal antibody (Abcam, 1∶1000). Nuclei were stained with DAPI. Arrowheads indicate a cluster of ICP0 speckles in the nucleus of two of the four neurons. DIC, differential interference contrast microscopy.
(TIF)

Figure S4 **Nuclear localization of the HCF-1 associated factors Ash2L and LSD1 in HeLa cells and unstimulated SCG neurons.** SCG-derived neurons were seeded onto glass coverslips and cultured for 7 days under conditions that support the establishment of HSV-1 latency before being fixed with 4% PFA. After quenching with 100 mM ammonium chloride, the samples were permeabilized with 0.1% Triton-X 100 and blocked using 1% BSA. Coverslips were incubated with either α-Ash2L (diluted 1∶1000) or α-LSD1 (diluted 1∶400) antibodies for 2 h followed by a fluorescent secondary antibody. HeLa cells served as a positive control and were seeded 1 day prior to fixation with 3.7% formaldehyde.
(TIF)
