# Supplementary figures and images for: Transient Reversal of Episome Silencing Precedes VP16-Dependent Transcription during Reactivation of Latent HSV-1 in Neurons
Source: PLoS Pathog. 2012 Feb 23;8(2):e1002540. doi: 10.1371/journal.ppat.1002540 (PMC3285597; doi:10.1371/journal.ppat.1002540)

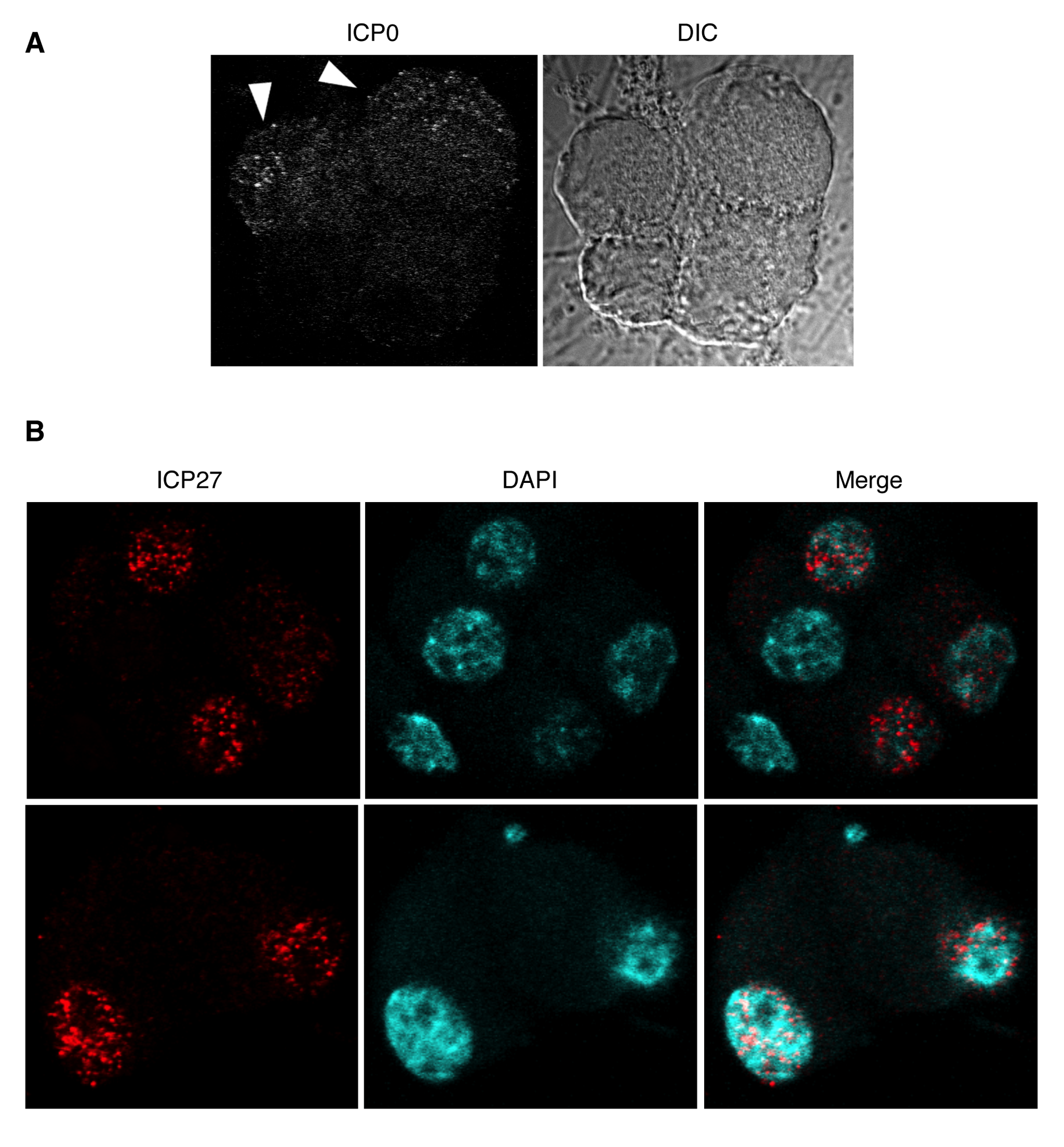

Supplement: Figure S3 — Lytic proteins ICP0 and ICP27 are expressed in Phase I and are localized to the nucleus. Week old SCG neuronal cultures were infected with either in1814 (A) or HSV GFP-Us11 (B) at MOI = 1 in the presence of ACV. After one week of establishing latency period, the cultures were induced with media lacking ACV but containing 20 µM LY294002. Samples were fixed either 25 hours (A) or 20 hours (B) after induction. ICP0 was detected using a monoclonal antibody (Virusys, 1∶100) and ICP27 was detected with a polyclonal antibody (Abcam, 1∶1000). Nuclei were stained with DAPI. Arrowheads indicate a cluster of ICP0 speckles in the nucleus of two of the four neurons. DIC, differential interference contrast microscopy. (TIF) [file ppat.1002540.s003.tif]

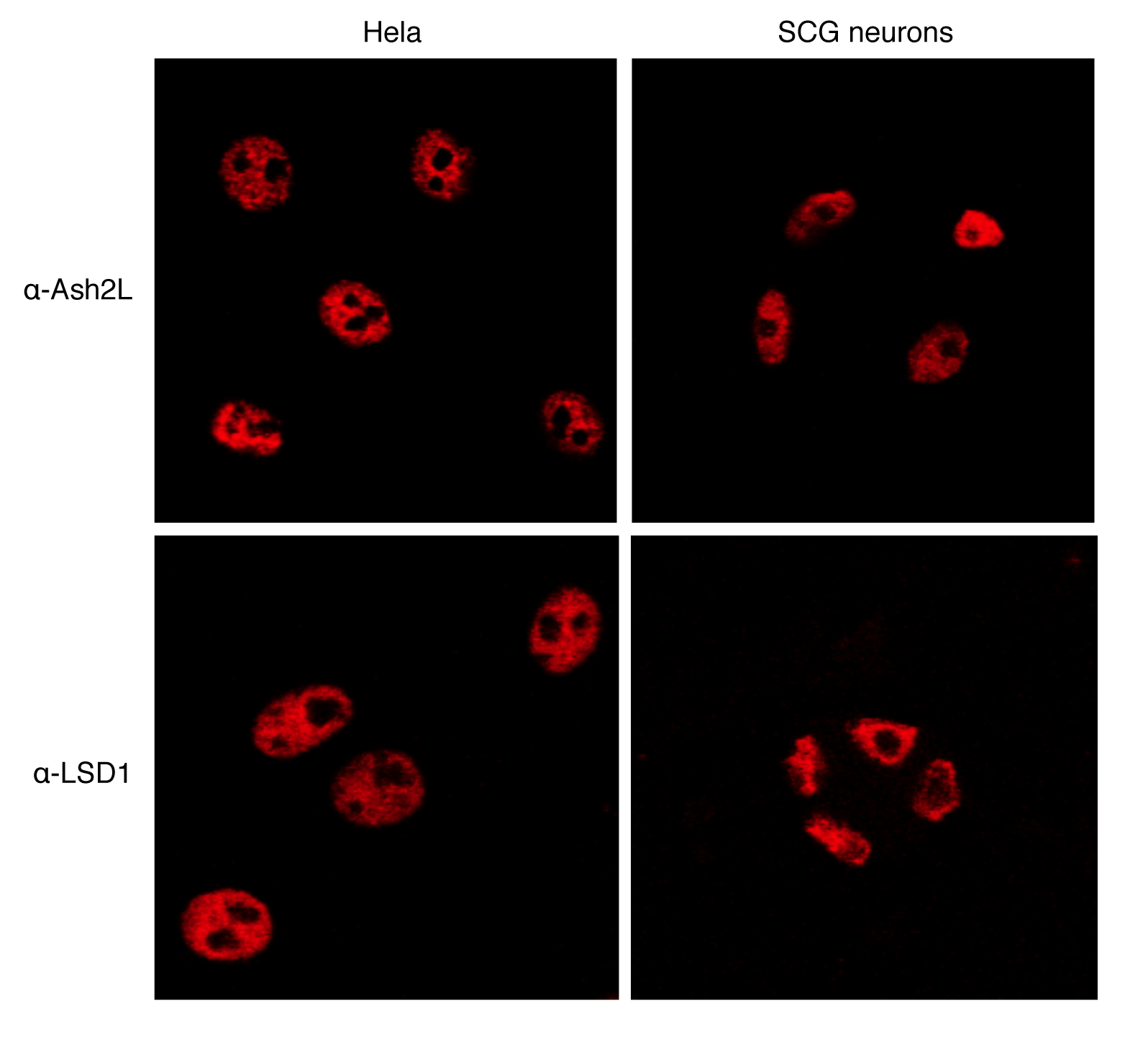

Supplement: Figure S4 — Nuclear localization of the HCF-1 associated factors Ash2L and LSD1 in HeLa cells and unstimulated SCG neurons. SCG-derived neurons were seeded onto glass coverslips and cultured for 7 days under conditions that support the establishment of HSV-1 latency before being fixed with 4% PFA. After quenching with 100 mM ammonium chloride, the samples were permeabilized with 0.1% Triton-X 100 and blocked using 1% BSA. Coverslips were incubated with either α-Ash2L (diluted 1∶1000) or α-LSD1 (diluted 1∶400) antibodies for 2 h followed by a fluorescent secondary antibody. HeLa cells served as a positive control and were seeded 1 day prior to fixation with 3.7% formaldehyde. (TIF) [file ppat.1002540.s004.tif]
